# Supplementary material for: Predicting complex phenotypes using multi-omics data in maize
Source: Plant Cell. 2026 Jun 15;38(6):koag185. doi: 10.1093/plcell/koag185 (PMC13318160; doi:10.1093/plcell/koag185)
Supplement: koag185_Supplementary_Data [file koag185_supplementary_data.zip › MS_6-8-26_Supplemental.pdf]

Supplementary materials

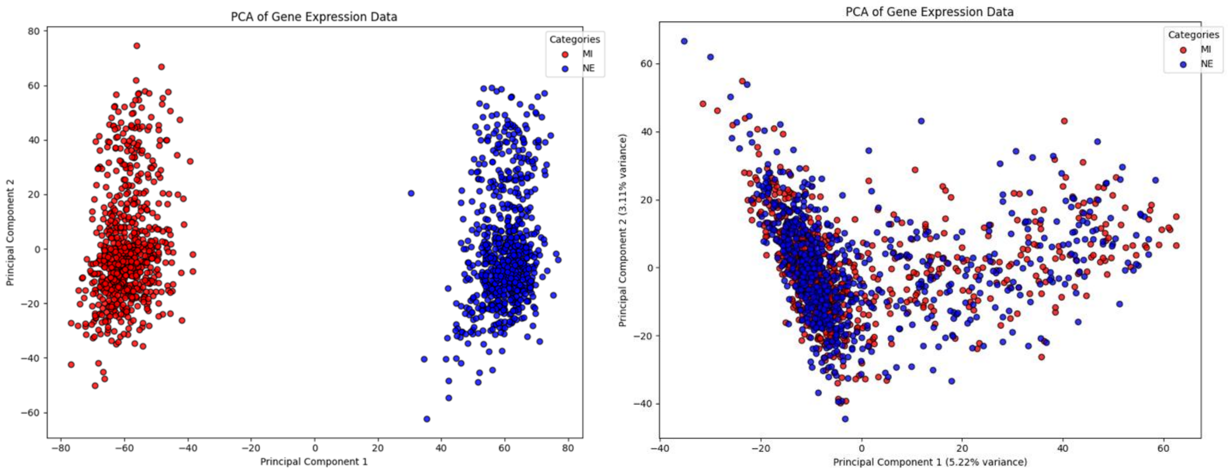

Supplemental Figure 1. Dimensionality reduction of gene expression input datasets pre and post batch correction. Principal component analysis (PCA) of the transcriptomic datasets. The left PCA is pre batch correction colored by field location. The right is post batch correction with field location set as the batch variable.

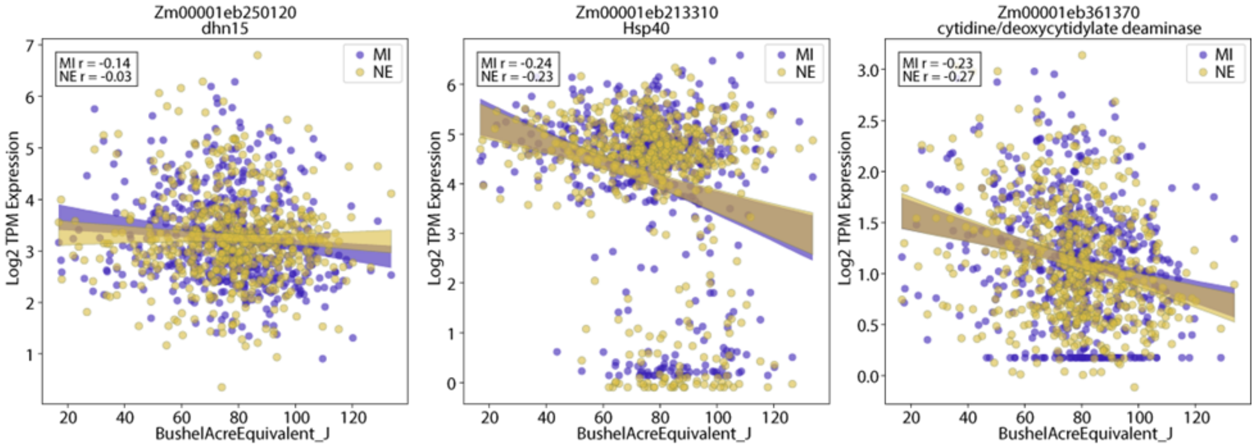

Supplemental Figure 2. Expression top features negatively associated with yield from the SVR transcriptome model. Scatterplots show the relationship between gene expression and bushels equivalent for a subset of top predictive features. For each gene, expression values from Michigan and Nebraska are plotted separately with fitted regression lines, and the Pearson correlation coefficient is shown for each environment.

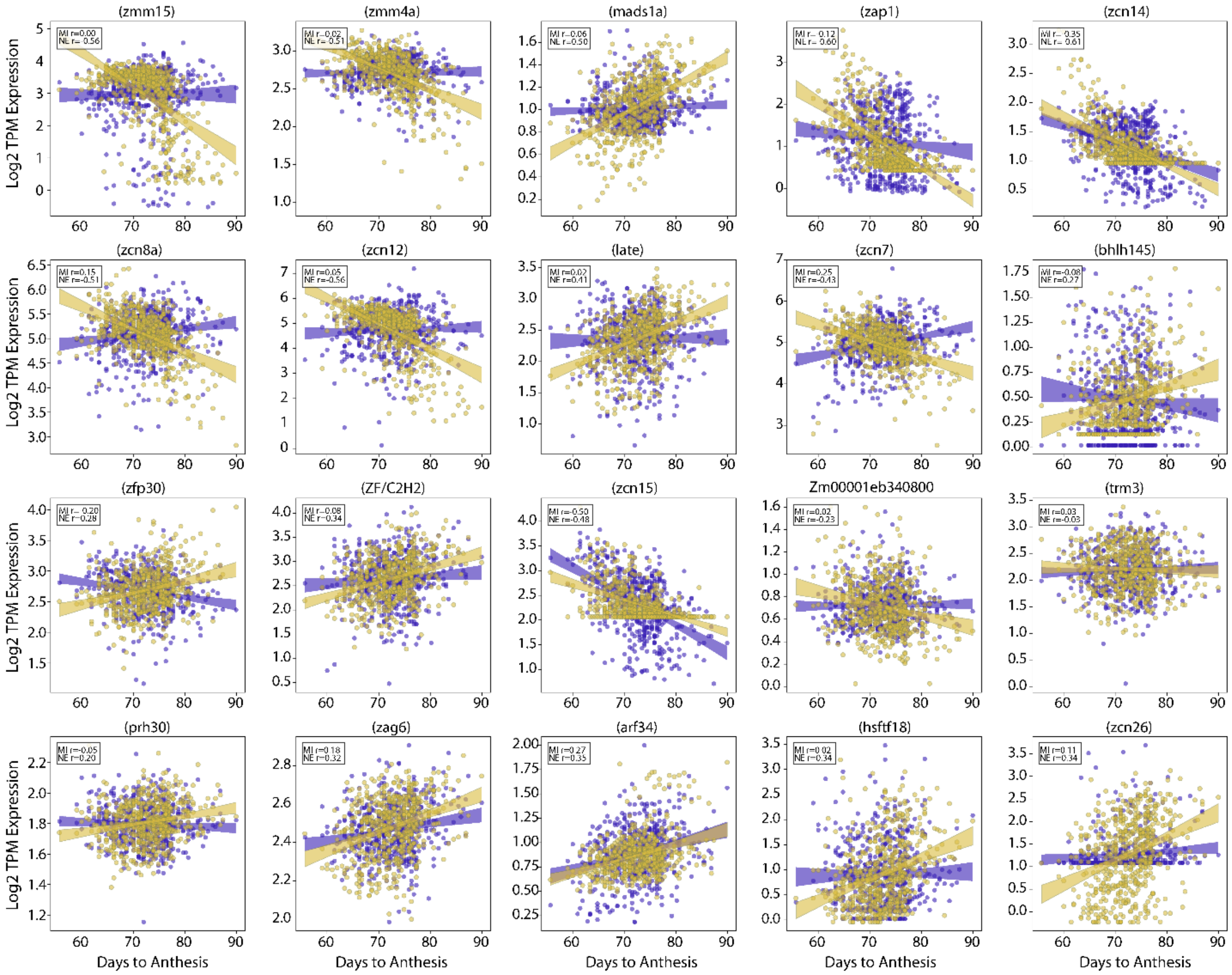

Supplemental Figure 3. Expression of flowering time genes across Michigan and Nebraska environments. Scatterplots show the relationship between gene expression and anthesis time for flowering time genes previously identified by transcriptome-wide association analysis in the Nebraska dataset. For each gene, expression values from Michigan and Nebraska are plotted separately with fitted regression lines, and the Pearson correlation coefficient is shown for each environment.

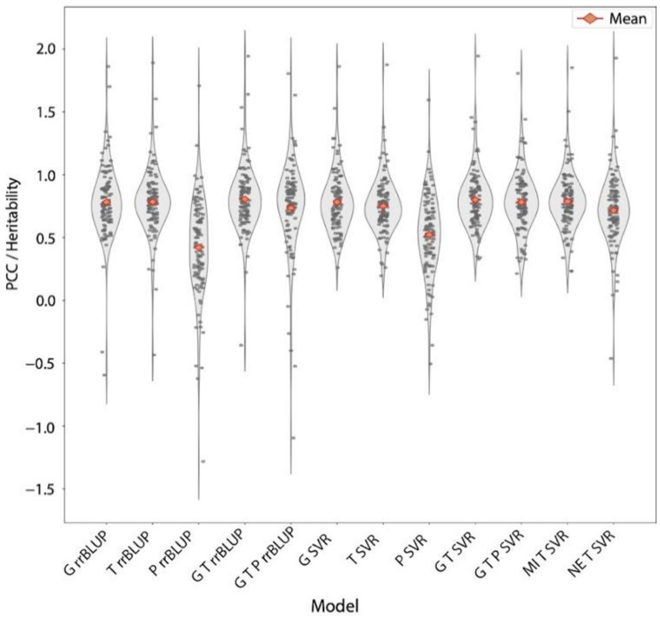

Supplemental Figure 4. Relationship between PCC and Heritability across input and model types. Violin plots of the PCC/h2 ratio plotted for every input and model type combination.

Gene Name/SNP	Feature Weight	Arabidopsis Ortholog	Related to Flowering Time
Zm00001eb214750 (zmm15 - Zea mays MADS-box 15)	-0.0323	AP1	Yes, flower development GO term
Zm00001eb283240	-0.0305	MIPS1	Inositol biosynthetic process
Zm00001eb266210 (LOC103630989)	-0.0274	N/A	Unknown
Zm00001eb07979 (dao1 - dioxygenase for auxin oxidation1)	-0.0260	ATDAO2	Auxin
Zm00001eb133230	0.0254	N/A	Hydrolase activity
Zm00001eb359020	-0.0252	N/A	Unknown
Zm00001eb234020 (aco6 - aconitase6)	0.0245	N/A	Metal ion binding
Zm00001eb337380	-0.0245	N/A	Unknown
Zm00001eb153190 (pebp12 - phosphatidylethanolamine-binding protein12)	-0.0244	TSF	Yes, regulation of flower development
Zm00001eb060540	-0.0243	N/A	ATP Binding

Supplemental Table 1. Highest weighted features from the best-performing model, SVR G + T for Days to Anthesis. Highest weighted features according to feature weight absolute value with their respective weight, Arabidopsis ortholog, and any related GO term.

Supplemental Table 2. Summary of phenotypic data used in this study. (see external excel file).

Supplemental Table 3. Vegetative Indices used in this study calculated from mean plot level reflectance values of the Red, Green, Blue, Near-infrared (NIR), or Red edge regions.

Index Name	Formula
**Normalized difference vegetation index - NDVI**	$NDVI = \frac{NIR - Red}{NIR + Red}$
**Green normalized difference vegetation index- GNDVI**	$GNDVI = \frac{NIR - Green}{NIR + Green}$
**Renormalized difference vegetation index - RDVI**	$RDVI = \frac{NIR - Red}{\sqrt{NIR + Red}}$
**Non-linear vegetation index - NLI**	$NLI = \frac{NIR^2 - Red}{NIR^2 + Red}$
**Modified Simple Ratio – MSR**	$MSR = \frac{\frac{NIR}{Red} - 1}{\sqrt{\frac{NIR}{Red}} + 1}$
**Chlorophyll vegetation index - CVI**	$CVI = \frac{NIR^2}{Green^2}$
**Normalized difference index - NDI**	$NDI = \frac{RedEdge - Red}{RedEdge + Red}$
**RedEdge normalized difference vegetation index - NDVI RedEdge**	$NDVI\ RedEdge = \frac{NIR - RedEdge}{NIR + RedEdge}$
**Plant senescence reflectance index - PSRI**	$PSRI = \frac{Red - Blue}{RedEdge}$
**RedEdge chlorophyll index – CI RedEdge**	$CIR\ RedEdge = \frac{NIR}{RedEdge} - 1$
**MERIS terrestrial chlorophyll index - MTCI**	$MTCI = \frac{NIR - RedEdge}{RedEdge - Red}$

Supplemental Table 4. Effect of hyperparameter tuning on SVR prediction accuracy for selected maize phenotypes.

Phenotype	PCC SVR Performance	PCC SVR Performance with Hyperparameter Tuning
Anthesis_A	0.74	0.76
ASI_L	0.11	0.002
Nodes_M	0.56	0.54
peri_N	0.39	0.36
RootArea1_O	0.33	0.24
